# Advances in Occurrence, Importance, and Mycotoxin Control Strategies: Prevention and Detoxification in Foods

**DOI:** 10.3390/foods9020137

**Published:** 2020-01-28

**Authors:** Sofia Agriopoulou, Eygenia Stamatelopoulou, Theodoros Varzakas

**Affiliations:** Department of Food Science and Technology, University of the Peloponnese, Antikalamos, 24100 Kalamata, Greece; estamatel@gmail.com (E.S.); theovarzakas@yahoo.gr (T.V.)

**Keywords:** mycotoxins, occurrence, detoxification, decontamination, foodstuffs, aflatoxins, fumonisins, ochratoxins, food safety, risk

## Abstract

Mycotoxins are toxic substances that can infect many foods with carcinogenic, genotoxic, teratogenic, nephrotoxic, and hepatotoxic effects. Mycotoxin contamination of foodstuffs causes diseases worldwide. The major classes of mycotoxins that are of the greatest agroeconomic importance are aflatoxins, ochratoxins, fumonisins, trichothecenes, emerging *Fusarium* mycotoxins, enniatins, ergot alkaloids, *Alternaria* toxins, and patulin. Thus, in order to mitigate mycotoxin contamination of foods, many control approaches are used. Prevention, detoxification, and decontamination of mycotoxins can contribute in this purpose in the pre-harvest and post-harvest stages. Therefore, the purpose of the review is to elaborate on the recent advances regarding the occurrence of main mycotoxins in many types of important agricultural products, as well as the methods of inactivation and detoxification of foods from mycotoxins in order to reduce or fully eliminate them.

## 1. Introduction

Mycotoxins belong to the category of toxic secondary metabolites, and they have a low molecular weight. They are produced by filamentous fungi belonging to the phylum Ascomycota or molds, and they have great importance in the health of humans and animals, being the cause of acute and chronic diseases [1,2,3,4]. Bennett defined that mycotoxins are natural products produced by fungi that induce a toxic response when introduced at a low concentration to higher vertebrates and other animals via natural route [5]. The Greek word “mykes” meaning “fungi” and the Latin word “toxicum” meaning “poison” are the origin of the word mycotoxin [6]. A variety of fungi such as *Aspergillus*, *Fusarium, Penicillium, Alternaria*, and *Claviceps* spp. colonize their host and produce mycotoxins [7]. Of the approximately 400 compounds identified as mycotoxins, 30 receive great attention, and they are considered a threat to human or animal health [8]. The most important mycotoxins are aflatoxins (AFs) (represented mainly by aflatoxin B1 (AFB1), B2 (AFB2), G1 (AFG1), G2 (AFG2), M1 (AFM1)), ochratoxins (OTs) (represented mainly by ochratoxin A (OTA)), fumonisins (FBs) (represented mainly by fumonisins B1 (FB1), B2 (FB2), and B3 (FB3)), trichothecenes (TCs) (with type A represented by HT-2 toxin (HT2) and T-2 toxin (T2), and type B represented mainly by deoxynivalenol (DON)), zearalenone (ZEN), the emerging *Fusarium* mycotoxins (fusaproliferin (FP), moniliformin (MON), beauvericin (BEA), NX-2 toxin, and enniatins (ENNs)), ergot alkaloids (EAs), *Alternaria* toxins (ATs) (such as altenuene (ALT), alternariol (AOH), alternariol methyl ether (AME), altertoxin (ALTs), and tenuazonic acid (TeA)), and patulin (PAT). Mycotoxins cannot be detected by eye, but they can be seen under ultraviolet (UV) light; moreover, they have no characteristic odor and they do not alter the organoleptic characteristics of foods [9].

Certain mycotoxins are produced by more than one fungal species, while some fungi are capable of producing more than one mycotoxin. Moreover, more than one mycotoxin can be found on an infected substrate [10]. Favorable climatic conditions cause more fungal and mycotoxin contamination in developing and tropical countries than in developed and temperate ones [11].

Two groups of fungi producing mycotoxins in food exist: field fungi that infect crops before harvest, and storage fungi which occur only after harvest. Among toxicogenic field fungi, three types can be distinguished: plant pathogens such as *Fusarium graminearum* (deoxynivalenol producer) and *F. verticillioides* (fumonisin producer), fungi that grow on senescent or stressed plants such as *Aspergillus flavus* (aflatoxin producer), and fungi which initially colonize the plant before harvest and predispose the commodity to mycotoxin contamination after harvest such as *Penicillium verrucosum* (ochratoxin producer) and *A. flavus* [1].

Various factors affect both the growth and the production of mycotoxins in many types of fungi, including temperature, humidity, environment, pH, water activity (a_w_), nutrients, level of inoculation, nature of the substrate, physiological state, and microbial interactions. This is why it is difficult for anyone to describe the set of optimal conditions for growth and production in physiological conditions [12]. Temperature 10–40 °C, pH 8.4, and a_w_ at levels above 0.70 are the conditions in which fungi usually develop [13]. Field fungi typically need 70%–90% relative humidity, a temperature of 20–25 °C, a_w_ > 0.85 for active growth, and a_w_ for optimal growth of 0.99. Active growth is the phase when the fungus grows at high rates in the mycelium. On the contrary, storage fungi are adapted to lower humidity and higher temperatures. Most *Aspergillus* and *Penicillium* species require a minimum of 0.75–0.85 a_w_ and grow well at 0.93–0.98 a_w._ *Aspergillus* species require a_w_ of 0.73 for active growth, while *Penicillium* species require a_w_ of at least 0.78–0.80. In addition, *Aspergillus* species adapt to temperatures of 30–40 °C, while *Penicillium* species exhibits good growth at temperatures of 25–30 °C [14].

Mycotoxins exist in agricultural commodities like peanuts [15], grapes and wines [3,16], grains [17,18], nuts, dried fruit, coffee, cocoa, spices, oil seeds, fruits, fruit juices, beer [14], and other foodstuffs and feed crops, both in the field and during transportation. At any stage of the food production process (before harvesting, harvesting, drying, and storage), fungal production of mycotoxins can occur and can expose consumers to the risk of contamination directly through food consumption or indirectly through feed [9]. In general, under prolonged storage conditions and at extreme temperatures along with extreme humidity, all crops including cereals can be subjected to mold growth and mycotoxin contamination [5]. The risk of producing mycotoxins increases with favorable conditions for fungal growth if bad farming and harvesting practices and inadequate drying, handling, packaging, storage, and transport conditions are applied [19].

Μycotoxicosis is the disease that results from exposure to mycotoxins (e.g., ergotism, alimentary toxic aleukia, aflatoxicosis), with effects on different organs of the human body, which can potentially cause death [9,20] and can be categorized as acute or chronic [1]. Effects in humans and animals following direct exposure to mycotoxins vary in terms of their toxicity, e.g., carcinogenic, endocrine disorders, teratogenic, mutagenic, hemorrhagic, estrogenic, hepatotoxic, nephrotoxic, and immunosuppressive [21]. Contact, ingestion, and inhalation are the main ways of exposing mycotoxins to the human body [6].

The control of mycotoxin contamination is based on two strategies: prevention of their production and detoxification [21]. Conventional cooking processes cannot destroy all mycotoxins. For partial or complete elimination of mycotoxins from food, several methods related to food processing, and numerous physical, chemical, and biological methods are applied [22].

The purpose of this review is to present an overview of the main mycotoxins, as well as their diversity in appearance and their importance for the health of humans and livestock. Moreover, control, prevention, and decontamination/detoxification strategies for food control and management are studied both before and after harvesting.

## 2. Occurrence and Importance of Mycotoxins in Foods

During different food processing technologies, including cooking, boiling, baking, frying, baking, and pasteurizing, most mycotoxins remain chemically and thermally stable. The result of contaminated feed is the presence of mycotoxins in animal foods such as meat, eggs, and milk, thereby leading to contamination of the human plate [3]. Regulatory limits on significant levels of mycotoxins in food and feed are established by various authorities worldwide such as the United States (US) Food and Drug Administration (FDA), the World Health Organization (WHO), the Food Agriculture Organization (FAO), and the European Food Safety Authority (EFSA) [5]. The International Agency of Research on Cancer (IARC) classifies some important mycotoxins into categories by examining the existence of sufficient human evidence for carcinogenicity, through toxicological studies [19]. Table 1 lists the major mycotoxins, their IARC number, the main producers, and some commonly contaminated foodstuffs, along with the US FDA and European Union (EU) regulatory limits for mycotoxin levels both in food and in animal feed [3,19].

**Figure 1:**
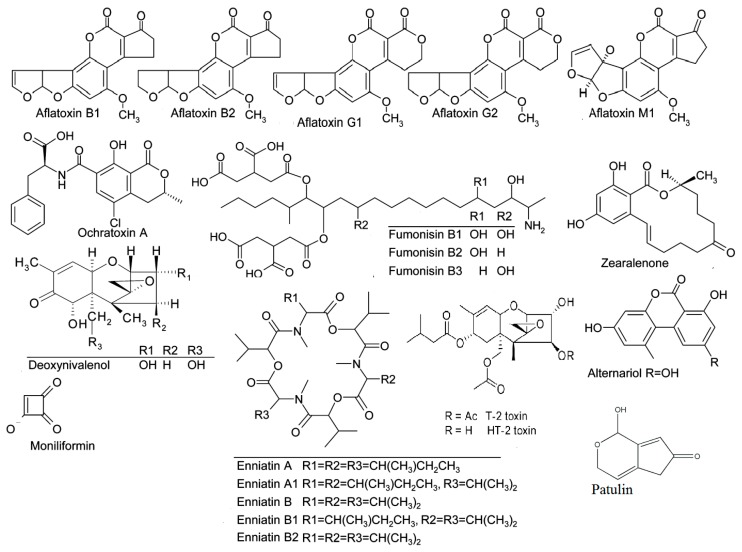
Chemical structures of the main mycotoxins.

The Rapid Alert System for Food and Feed (RASFF) monitors the contamination of food and feed by mycotoxins on a weekly basis in Europe. Through RASFF, all EU member states can be informed via an information exchange system and take measures to ensure the safety of food and feed [14]. Mycotoxins consistently constitute the highest risk category for notifications, and, every year, they are found among the “top ten” hazards reported annually by the RASFF. The records of the decade 2009–2018 [23,24,25,26,27,28,29,30,31,32] show that aflatoxins held the highest percentages (Table 2), while, based on the latest RASFF report for the year 2018 in the EU (Table 3), mycotoxin notifications amounted to 655, with aflatoxin notifications totaling 536, accounting for a significant proportion (82%) [32]. Figure 1 presents the chemical structures of the main mycotoxins.

The agricultural industry has to deal with the presence of mycotoxins in food, as it is of global importance and a major threat [33]. In developing countries, factors such as poor food quality control, hot climate, poor production technologies, and poor crop storage conditions favor the development of fungi and the formation of mycotoxins, resulting in the more frequent occurrence of mycotoxin-contaminated foods in these countries [34]. Huge agricultural and industrial losses in billions of dollars occur annually because 25% of the world’s harvested crops are contaminated by mycotoxins [19]. The increased cost of production, the lowered animal production, the decreased market values, the irregularity of production [35], the regulatory enforcement, and the testing and other quality control measures [9] are some of the significant sources of economic loss due to the occurrence of mycotoxins in food and feed.

In the recent press release of the IARC and WHO in 2016, calls for action on mycotoxin contamination in developing countries were undertaken because, according to the report, on a daily basis, 500 million people in developing countries who are exposed to financial burdens are exposed to natural toxins, including mycotoxins, and, globally, 160 million children from developing countries under the age of five are stunted [36]. A search for the occurrence of major mycotoxins was made based on research in Scopus, ScienceDirect, Google Scholar, and Web of Science, between 2014 and 2019.

### 2.1. Aflatoxins

Aflatoxins are secondary metabolites, and they belong to the category of difuranocoumarins [44]. Under warm and humid conditions, *Aspergillus flavus, A. nomius*, and *A. parasiticus* produce AFs [22,45], commonly found in food and feeds. The species *A. flavus* and *A. parasiticus* are found worldwide in the soil and in the air [46], preferring to grow at temperatures between 22 and 35 °C and a_w_ between 0.95 and 0.98 [47]. Other species producing aflatoxins similar to *A. flavus* are *A. zhaoqingensis* and *A. bombycis*, while those similar to *A. parasiticus* are *A. toxicarius* and *A. parvisclerotigenus*. Moreover, *A. pseudotamarii*, *A. ochraceoroseus*, *A. rambellii*, *A. toxicarius*, *Emericella astellata*, *E. olivicola*, and *E. venezuelensis* are some species of mycotoxin producers. Τwo other recently described aflatoxigenic species are *A. minisclerotigenes* and *A. arachidicola* [48].

AFs are the best known among all mycotoxins, because of their serious impact on human and animal health. Four main types of aflatoxins are the most studied among more than 20 known ones; these are aflatoxin AFB1, AFB2, AFG1, and AFG2, named after the fluorescence they display in UV light (B for blue and G for green). The hydroxylated metabolites of AFB1 and AFB2 are aflatoxin M1 (AFM1) and aflatoxin M2 (AFM2), which are present in the meat of animals that consumed aflatoxin-contaminated feed, as well as animal products such as eggs, milk, and cheese [22]. Aflatoxin B1 is a carcinogenic substance (according to the classification by the IARC in 1987) (category 1A), while AFM1 is a potentially carcinogenic substance (category 2B) [49], with a toxicity range of B1 > G1 > B2 > G2 [50]. AFB1 is considered to be the most potent carcinogenic toxin known in mammals [51], and food contamination should be reduced to the lowest possible level, since no food or health organization established a tolerable daily intake for humans (tolerable daily intake, TDI) [52]. Exposure to chronic hepatitis B virus infection and aflatoxin may increase liver cancer risk by up to 30 times compared to the risk in individuals exposed to aflatoxin only [53]. The risk of exposure to contaminated foods with varying levels of AFs worldwide exists for more than 4.5 billion people [54]. At present, levels of AFs in food and feed are established in approximately 100 countries [55]. The EU legal limit for AFB1 in processed cereal foods is 0.02 µg/kg [56]. Different maximum upper limits are set worldwide for AFM1 in milk or milk products, with Codex Alimentarius and the EU setting the limit to 0.05 µg/kg AFM1, whereas the US and some Latin American countries set it to 0.5 µg/kg [57].

Aflatoxins are the first mycotoxins to be initially classified as toxic, following research into the deaths of 100,000 poultry (mainly turkeys) in England. Originally, the causes of the strange disease at that time were unknown; thus, the disease was named “X disease “, that is, turkey X disease. It was later found that the cause was the growth of *A. flavus* in poultry feed. This led to a breakthrough in the research on the field of mycotoxins, which in turn led to the intensive and systematic checks on any moldy product and setting maximum limits [9].

Pre-harvest and post-harvest factors are related to the production of AFs. Thus, pre-harvest weather conditions associated with periods of drought and heat stress during flowering and fruit growth were reported to be the main factors responsible for the increased infection with AFs produced by *A. flavus* and *A. parasiticus* in maize, pistachio, cotton, and nuts [58]. Furthermore, other stress factors in plants, such as inadequate nutrition, insect nutrition from growing fruits, weed competition, overgrowth of plants, and plant diseases, facilitate fungal infection and the production of AFs [58]. After harvesting, higher concentrations of AFs are observed due to improper storage of the products, such as storage with inadequate moisture content and inappropriate temperature. If the product is quickly dried and stored under appropriate conditions, and the a_w_ value does not exceed 0.78, aflatogenic molds do not grow well. The biosynthesis of AFs is inhibited at an a_w_ of less than 0.8334. Because the production of AFs depends on the a_w_ interaction with temperature, maintaining the temperature in the storage area below 15 °C leads to minimum a_w_ for the production of mycotoxins at 0.934. Moreover, the formation of AFs can cause damage to the products [59].

Aflatoxins are linked to various diseases, such as aflatoxicosis, in animals, pets, and humans around the world [44], and they are considered to be particularly harmful as they have carcinogenic, mutagenic (DNA damaging), teratogenic, and immunosuppressive effects [51]. Symptoms of acute aflatoxicosis in humans include vomiting, abdominal pain, jaundice, pulmonary edema, coma, convulsions, and death [5,60], while chronic aflatoxicosis occurs via cancer, immune system inhibition, and liver damage. There are significant differences in species sensitivity, with the size of the reaction depending on a variety of factors, such as age, sex, weight, nutrition, metabolism, exposure to infectious agents, and the occurrence of other mycotoxins [5], as well as the type of toxin, mechanism of action, and levels of intake [61]. In many areas of the world, where liver cancer occurs in large numbers in the population (e.g., in southeast Asia and sub-Saharan Africa), chronic hepatitis C infection and aflatoxin exposure are considered important risk factors, since they are likely to interact synergistically [62].

In India, the most serious outbreak of human hepatitis was recorded in 1974, when 108 of 397 patients died after consuming heavily contaminated maize with AFs at levels of 0.25–15 mg/kg [63], while the largest and most serious case of acute aflatoxin poisoning in humans worldwide, recorded in April 2004 in Kenya, resulted in 125 out of 317 patients losing their lives (mortality rate, 39.4%) after eating infected maize, with aflatoxin levels of 5–20 mg/kg [64]. A smaller-scale epidemic occurred in Kenya in 2005, causing 16 deaths [65], while, in the same country in 1981, 12 deaths were recorded from consumption of contaminated maize at levels of 3.2–12 mg/kg with AFB1 [64]. In addition, encephalopathy and visceral degeneration in children are symptoms of Reye’s syndrome, which is linked to aflatoxin toxicity [19].

Aflatoxin contamination was reported in various countries such as Argentina [66], Brazil [67], China [68], Italy [69], Portugal [70], Spain [71], and Tanzania [72]. In food analysis, the presence of AFB1 is often the highest in the AF mixture [19]. AFs are mainly detected in cereals (barley, corn, rice, wheat, oat) [33] and their derivatives (bread, flour, breakfast products, cornflakes and pasta) [4], in nuts (almonds, pecans, pistachios, walnuts, cashews, and Brazil nuts) and peanuts [73], in species and herbs [6,74], in edible vegetable oils [55], in wines [75], in sugarcane [76], in cottonseed [40], in dried fruits [77], and in animal food products such as milk [78], eggs [79], cured meat [80], and animal tissues [81]. Some detailed recent studies on AF occurrence in foods are presented in Table 4. The problem of AFs is very important around the world and particularly in Africa, where aflatoxin contamination is reported in raw cereals with 50% incidence, with infestation reaching 1642 μg/kg in rice [33].

### 2.2. Ochratoxin A

Among the ochratoxin categories A, B, and C, OTA is the most abundant and harmful mycotoxin that contaminates foods [93]. OTA was first identified in South Africa, from the fungus *A. ochraceus*, from which it derives its name. It is chemically known as the phenylalanyl derivative of a substituted iso-coumarin (*R*)-*N*-[5-chloro-3,4-dihydro-8-hydroxy-3-methyl-1-oxo-1*H*-2-benzopyran-7-y1] carbonyl]-l phenylalanine [94]. *Aspergillus* and *Penicillium* are the two main genera of OTA producers. The main producing species belong to the *Aspergillus* section *Circumdati*, *Aspergillus* section *Nigri*, *P. verrucosum*, *P. thymicola*, and *P. nordicum* [19]. The non-chlorinated analogue, ochratoxin B, which is much less toxic, sometimes co-occurs with OTA in food and feed [53]. Although OTA produced by *Aspergillus* can likely occur pre-harvest, recent studies [95] pointed to OTA in grains as mainly a storage issue.

Ochratoxin is linked to immunotoxic, genotoxic, neurotoxic, carcinogenic, nephrotoxic, and teratogenic effects, considered the most toxic ones among the ochratoxin family members. Moreover, it is classified by the IARC as a possible human carcinogen (Group 2B) [20], but the specific mechanism of toxicity is not fully understood. Increased incidence of testicular cancer in animals is associated with ingestion of OTA [19]. Although OTA is liable to decomposition in the rumen, it was found in cow’s milk [96]. The mutagenic capacity of AFB1 could be increased in cases of co-occurrence with OTA in some crops [97]. A provisional tolerable weekly intake (PTWI) of 112 ng/kg body weight (b.w.) was proposed by the Joint FAO/WHO Expert Committee on Food Additives (JEFCA) [98].

Ochratoxin production is observed in the a_w_ range of 0.92–0.99, with the maximum concentration being in the range 0.95–0.99 depending on the strains. The optimum temperature for OTA production is 20 °C, followed by the temperature of 15 °C, with significantly lower production at 30–37 °C [99]. Taking into account that *Aspergillus* and *Penicillium* responsible for the production of OTAs have a temperature range of 12–37 °C for *A. ochraceus* and 0–31 °C for *P. verrucosum*, OTA can be produced in all agricultural areas of the world [33].

Ochratoxin was reported in cereals [28], in species [6], in alcoholic beverages such as in wines [75] and in beer [100], in dried vine fruits [40], in coffee [101], in cocoa and chocolate [102,103], in meat [104], and in milk [96]. Among foods, cereals occupy the first position of the total exposure to OTAs with 60% [105]. The maximum limits of 5 ng/g OTA in raw cereal grains, 3.0 ng/g in cereal-processed products, 10 ng/g in coffee and dried fruits, 2 µg/L in wine, and 0.5 ng/g in cereal-based baby foods are set by European commission (EU) [106].

Pig’s blood, kidney, liver muscle, and adipose tissue are some of the tissues where OTA was detected with rather high levels found in animals suffering from porcine nephropathy, especially in countries of the Balkan Peninsula [53]. In a human disease of kidney referred to as Balkan endemic nephropathy, OTA is implicated. The disease is characterized by tubule interstitial nephritis, and OTA is associated with a high incidence of kidney, pelvis, ureter, and urinary bladder tumors in some eastern European countries [51]. It is also suspected that the inhalation of OTs via air and dust caused by the opening of Egyptian tombs may have led to the deaths of archaeologists [6].

Recent OTA studies in food commodities are presented in Table 5.

Risk assessments were carried out based on OTA occurrence data in Brazil [118], Benin, Cameroon, Mali, Nigeria [119], and Paraguay [103]. According to these studies, the majority of the population did not exceed the TDI.

The impact of OTA in recent research shows the importance of reinforcing OTA control strategies in food production. Although mycotoxins are extremely stable during food processing, there are several factors that can affect their stability [6]. At different stages of food processing such as baking, roasting, frying, brewing, canning, and peeling, OTA cannot be completely deactivated [120]. Moreover, it was reported that OTA can be transferred into beer and wine samples from contaminated grains [121]. Feeding animals with OTA-infected bread can cause its accumulation in the meat of animals intended for human consumption [122].

### 2.3. Fumonisins

Fumonisins belong to a large group of toxins referred to as *Fusarium* toxins, which occur in cereals originating from pathogenic fungi, mostly *Fusarium verticillioides* and *Fusarium proliferatum* [123]. In addition, *Aspergillus niger* can produce fumonisins on grapes and raisins [124]. The group of 28 analogues of FBs is divided into four main groups: fumonisin A, B, C, and P. In addition to A, B, C, and P, there is also Fumonisins “Py” that can occur [123]. The fumonisin B (FB) analogues, which include FB1, FB2, and FB3, occur in nature with the highest frequency, whereas FB1 is usually found at the highest concentrations [125]. Fumonisins cause health effects in animals, especially in the liver and kidney, although data for the health effects of fumonisins in humans remain limited [126]. FB1 can cause leukoencephalomalacia in horses [127], and pulmonary edema syndrome and hydrothorax in pigs [128].

The IARC classifies FB1 and FB2 as possibly carcinogenic to humans (Group 2B) [20], and the JECFA set a provisionally maximum tolerable daily intake (PMTDI) of 2 µg/kg b.w./day for FB1, FB2, and FB3 alone or in combination [129]. Acceptable upper limits of 800–4000 and 2000–4000 µg/kg FB1 and FB2, respectively, were set by the European Union [38] (EU Regulation 1126/2007) and the US, in cereal-based products [130].

Fumonisins contaminate cereals [131] and their derivatives [132,133], and they also exist in maize and maize-based products [134], asparagus [135], grapes, and raisins [124]. Table 6 presents representative studies on the occurrence of FUs (μg/kg) in food samples worldwide during 2014–2019.

### 2.4. Trichothecenes

Trichothecenes are produced by different species of *Fusarium*, *Myrothecium*, *Trichoderma*, *Trichothecium*, *Cephalosporium*, *Verticimonosporium*, and *Stachybotrys* [53]. These cyclic sesquiterpenoid toxins are characterized by a variable number of acetoxy and hydroxyl groups, an epoxide ring at position C_12_-C_13_, and a double bond between C_9_ and C_10_ [141]. TCs are easily absorbed into the gastrointestinal membranes and are rapidly distributed to various organs and tissues of the body because of their low molecular weight and amphipathic nature [40]. More than 200 different TCs are known at present, subdivided into four basic groups: A, B, C, or D. The most toxic groups of trichothecenes are type A (T-2 and HT-2) compared to type B analogues (e.g., nivalenol (NIV), DON, and fusarenon-X (FX)) [142].

#### 2.4.1. Trichothecenes Type A (HT-2 Toxin and T-2 Toxin)

The main fungal species producing HT-2 and T-2 toxins is *Fusarium langsethiae.* *Fusarium poae, Fusarium sporotrichioides, F equiseti,* and *F. acumninatum* were also found to produce T-2 and HT-2 [143,144].

HT-2 and T-2 toxins are found in oat, barley, wheat, maize, and rice, as well as in cereal-based products and soybean [53,98]. HT-2 and T-2 toxins show toxic effects such as growth retardation, myelotoxicity, hematotoxicity, and necrotic lesions on contact sites [145]. A TDI of 100 ng/kg b.w./day for T-2 and HT-2 toxins was set by the EFSA [144].

#### 2.4.2. Trichothecenes Type B (Deoxynivalenol)

Deoxynivalenol, also denoted as vomitoxin, is produced by fungi of the *Fusarium* genus, mainly by *Fusarium graminearum* and *F*. *culmorum*, which are both associated with FHB disease (*Fusarium* head blight) in cereals, especially oats, barley, wheat rye, and maize, and less frequently in rice, sorghum, and triticale [17,146]; it also contaminated cereal-derived food products such as bread, pasta, or beer. DON is the predominant trichothecene all over the world, although it is among the least toxic of the trichothecenes, and its occurrence is considered to be an indicator of the potential presence of other, more toxic trichothecenes [53]. DON is the most frequently detected mycotoxin in cereal grains worldwide. The average incidence is 59%, with 50% and 76% in Asia and Africa, respectively, with a tendency for higher concentrations in Europe and Asia [33]. Moreover, levels of DON in grains (wheat, maize, and barley) in North America do not appear to be different from those reported around the world, and they can be much higher than usual in certain years due to more severe *Fusarium* infection [147].

The maximum level of DON of 200 µg/kg was set by the EU in cereal-based baby foods for infants and young children, as well as levels of 750 µg/kg in flour and 500 µg/kg in breads [146]. At high temperatures, DON is a very stable trichothecene (120 °C, moderately stable at 180 °C), as well as soluble in water and in polar solvents such as aqueous methanol, acetonitrile, and ethyl acetate [148], and it is found during the storage and processing of products [100]. The DON effect of protein synthesis inhibition is through binding to the ribosomal subunit, leading to ribotoxic stress [149]. The most common route of exposure to DON is through food. Acute gastrointestinal symptoms such as vomiting, hemorrhagic diarrhea, and refusal of food may occur in animals after ingestion of highly contaminated animal feed. Symptoms such as anorexia, suppression of body weight gain, hepatotoxicity, dermatological problems, and altered nutritional efficacy appear after long-term dietary exposure to DON. The acute effects of DON on animals and humans are similar [17]. Consumption of DON-contaminated cereals was linked to acute gastroenteritis and emesis in India [2]. The mutagenic and/or carcinogenic properties of DON are not established by experimental or epidemiological evidence and, therefore, the IARC classifies DON as not carcinogenic to humans (Group 3) [49]. The FAO/WHO and JECFA established a PMTDI of 1 mg/kg b.w. for the sum of DON and its acetyl derivatives (3-ADON and 15-ADON) [150]. A PMTDI of 1 μg/kg b.w./day for DON and its metabolites, 3-ADON, 15-ADON, and DON-3-glucoside (DON-3G), was set recently by the EFSA [151].

The occurrence of DON is reported in various countries such as Paraguay [100], Lebanon [74], Nigeria [152], China [148], Tunisia [153], Croatia [154], and Morocco [155]. Representative studies on the occurrence with DON, HT-2, and T-2 are summarized in Table 7. Studies included raw cereals (wheat, maize, rice, barley, oat), flour, bakery products, and infant foods. DON incidence ranged from 3% in flour to 100% contamination of wheat grains, bakery products, and oats. DON contamination in infected grains is associated with the severity of FHB disease in the field. FHB control in the field during cultivation is the best way to control DON [156].

### 2.5. Zearalenone

The fungi of the *Fusarium* genus produce ZEN; in particular, fungal species of *F*. *graminearum* (*Gibberella zeae*), *F*. *culmorum*, *F*. *crookwellense, F*. *semitectum*, and *F*. *equiseti* are the major producers of ZEN, infecting cereals and food worldwide, mainly in temperate climates [1,183]. While contamination with ZEN is low in grains in the field, it increases in storage conditions with moisture of more than 30%–40% [1]. Currently, the limits for ZEN in cereals vary between countries and range from 50 to 1000 µg/kg [184]. A TDI for ZEN of 0.25 μg/kg b.w./day was established by the EFSA [40]. Moreover, maximum permissible limits for ZEN should be within the range of 100–200 μg/kg in unprocessed cereals, 75 μg/kg for processed cereals, 20 μg/kg in processed cereal foods, and 50 μg/kg in cereal snacks according to EU legislations [185]. Risk assessments were performed on the basis of ZEN exposure data in France, Germany, Finland, China, and India. 

In only a few cases, the possible ZEN intake was found to exceed the TDI, and almost all studies agreed that the majority of the population did not exceed the TDI value given by the EU [183]. ZEN is soluble in aqueous alkali, acetone, acetonitrile, benzene, methyl chloride, alcohols, and ethers, but insoluble in water [98]. The IARC classifies ZEA as a Group 3 carcinogen [49]. ZEN is a non-steroidal estrogenic mycotoxin and works by mimicking the effects of the female estrogen hormone, affecting conception, ovulation, and fetal development at concentrations above 1 mg/kg [166]. ZEN can lead to hyperestrogenism, mainly affecting reproduction. The most susceptible species to ZEN infection are prepubertal swine. Swelling of the vulva, increases in uterine size and secretions, mammary gland hyperplasia and secretion, prolonged estrus, anestrus, increased incidence of pseudopregnancy, infertility, decreased libido, and secondary complications of rectal and vaginal prolapses, stillbirths, and small litters are some of the typical clinical symptoms of hyperestrogenism [1].

Occurrence of ZEN is reported both in various developed countries like Germany [186], and Japan [187] and in developing countries like Egypt [188], Thailand [189], Iran [190] Croatia [154], and the Philippines [161]. It is worth mentioning that zearalenone is a synthetic nonsteroidal estrogen of the resorcylic acid lactone group related to mycoestrogens found in fungi in the *Fusarium* genus, and it is used mainly as an anabolic agent in veterinary medicine, where it can also contribute to related exposure/toxicity [191]. Table 8 presents representative studies on the occurrence of ZEN (μg/kg) in food samples worldwide during 2014–2019.

### 2.6. Emerging *Fusarium* Mycotoxins (Fusaproliferin, Moniliformin, Beauvericin, NX-2 Toxin, and Enniatins)

In recent years, emerging mycotoxins became a major issue due to their high occurrence in cereals and their products [120]. In a more recent paper, emerging mycotoxins were defined as “mycotoxins, which are neither routinely determined, nor legislatively regulated; however, the evidence of their incidence is rapidly increasing” [195]. Currently, an opinion on the presence of ENNs and BEA in food and feed was reported by the EFSA without a risk assessment due to the lack of relevant toxicity data. Moreover, until now, there are no maximum levels for emerging *Fusarium* mycotoxins [196].

Fusaproliferin is a bicyclic sesterterpene produced by *Fusarium* species such as *F. proliferatum F. subglutinans*, and *F. verticillioides* [197]. FUs showed toxicity on chicken embryos and brine shrimp larvae [197].

Structurally, MON is a 1-hydroxycyclobut-1-ene-3,4 dion, a small molecule, soluble in water, which can be produced by *F. verticillioides*, *F. begoniae*, *F. denticulatum*, *F. lactis*, *F. nisikadoi*, *F. phyllophilum*, *F. pseudocircinatum*, *F. pseudonygamai*, *F. ramigenum*, *F. tricinctum*, *F. acutatum*, *F. anthophilum*, *F. bulbicola*, *F. concentricum*, *F. diaminii*, *F. fujikuroi*, *F. napiforme*, *F. nygamai*, *F. proliferatum*, *F. pseudoanthophilum*, *F. sacchari*, *F. subglutinans*, *F. thapsinum*, *F. beomiforme*, *F. oxysporum*, *F. redolens*, *F. chlamydosporum*, *F. arthrosporiodes*, *F. avenaceum*, and *F. acuminatum* [198], and recently proven to be a metabolite of *Penicillium melanoconidium* [197].

Structurally, BEA is cyclic hexadepsipeptide consisting of an alternating sequence of three d-a-hydroxy-*iso*-valeryl- and *N*-methyl-l-phenylalanyl residues [199]. BEA was first isolated from *Beauveria bassiana*, a fungus that causes diseases in insects [199], but it is frequently found in corn and corn-based foods and feeds infected by *Fusarium* spp. BEA occurs in cereal and cereal-based products not only in different European countries such as Romania [200], Spain [201], Italy [112], and Czech Republic [92], but also throughout the world in countries such as Japan [202], Tanzania, Rwanda [203], Iran [204], and Morocco [205]. BEA has antibacterial, antifungal, and insecticidal activities and causes toxic effects such as induction of apoptosis, increased concentration of cytoplasmic calcium, and DNA fragmentation in mammalian cell lines [199].

A new trichothecene mycotoxin, named NX-2, was recently characterized in rice cultures. NX-2 is similar in structure and similar in toxicity to 3-ADON, but lacks the keto group at C-8; hence, it is a type A trichothecene [206].

The *Fusarium* species identified as producers of ENNs are *F. merismoides*, *F. acuminatum*, *F. arthrosporioides*, *F. avenaceum*, *F. compactum*, *F. culmorum*, *F. equiseti*, *F. kyushuense*, *F. langsethiae*, *F. lateritium*, *F. oxysporum*, *F. poae*, *F. sambucinum*, *F. scirpi*, *F. sporotrichioides*, *F. torulosum*, *F. tricinctum*, and *F. venenatum* [207]. Fusarium species capable of producing ENNs can be found in different geographical areas, and the extent of seed contamination is only occasionally as high as mg/kg [208]. ENNs contaminate not only cereal grains but also many kinds of foods including vegetable oil, beans, dried fruits, tree nuts, and coffee [196]. The ENNs most detected in foods and feed are enniatin A, (ENA), enniatin A1 (ENA1), enniatin B (ENB), and enniatin B1 (ENB1) [209]. With regard to enniatins, there is relatively little to indicate that it is of concern to humans and animals; however, it may play a role in pronouncing the impact of other *Fusarium* toxins (i.e., DON) by inhibiting cellular export [210].

Due to their high prevalence in feed and food, possibly at high concentrations, as well as their potential toxicity to animals and humans, research interest in emerging mycotoxins increased [199]. Table 9 presents representative studies on the occurrence of emerging *Fusarium* mycotoxins (μg/kg) in food commodities worldwide during 2014–2019.

### 2.7. Ergot Alkaloids

The EA group of mycotoxins is derived from the genus *Claviceps*, which is a phytopathogen, with effects known from the Middle Age (ergotism, the human disease historically known as St. Anthony’s Fire) [42], and it is classified as a tryptophan-derived alkaloid [213]. Fungal structures of *Claviceps* species are produced instead of kernels on grain ears or seeds on grass heads, with large and dark sclerotia representing the final stage of the disease, known as “ergots” [213]. Psychological and physiological effects in humans can occur by ergot poisoning, affecting blood supply to the extremities and central nervous system, while, in animal health, there are problems associated with reduced productivity, diarrhea, and internal bleeding [44,214].

In Europe, a series of EAs, such as ergocryptine, ergocristine, ergotamine, ergosine, ergometrine, ergocornine, and their corresponding epimers can be detected in the sclerotia after the contamination of *Claviceps purpurea,* which is the major producer of EAs [68]. *C. purpurea* is widespread throughout the world and infects many monocotyledonous plants, like cereal grains and forage grasses [42,215]. Main affected crops by EAs are cereals like rye, barley, wheat, millet, oats, and triticale [216]. Among cereals, rye has the highest rates of fungal contamination by *C. purpurea* as it is a cross-pollinator with large open florets [217]. As a result, ergot alkaloids appear more in rye and rye-based foods as compared to other cereals [217]. Even if ergot bodies are removed by a hand-cleaning procedure, EAs could remain in grains [214].

Ergocristine, ergosine, ergotamine, ergometrine, ergocornine, and ergocryptine are the EAs with the highest frequency of detection [214]. In the EU, no maximum permitted levels of EAs are set for feed or food, and the only available standard (EU) sets a limit of 0.5 g/kg for the sum of ergot alkaloids in unprocessed cereals, except for maize and rice [218]. Based on toxicological studies on their vasoconstrictive effects and following an estimate of human and animal dietary exposure by the EFSA, a group TDI of 0.6 µg/kg b.w./day was derived [213]. The maximum permissible level of 300 mg of ergot bodies/kg grain was set by the United States and Canada, while 0.01% is the limit in China of the total ergot alkaloid content in cereals [217]. Table 10 presents representative studies on the occurrence of EAs (μg/kg) in food samples worldwide during 2014–2019.

### 2.8. Alternaria Toxins (Altenuene, Alternariol, Alternariol Methyl Ether, Altertoxin, Tenuazonic Acid, Tentoxin)

The *Alternaria* species can be found everywhere and in many ecosystems such as plants, seeds, agricultural commodities, atmosphere, and soil [220]. They produce *Alternaria* toxins that contaminate foods in storage [221], with AOH, AME, TeA, ALT, ATXs, and TeA being the most important. More than 70 secondary metabolites are produced by the toxin-producing *Alternaria* [222], including species such as *A. alternata*, *A. brassicae*, *A. dauci*, *A*. *japonica*, *A. solani*, *A. tenuissima*, and *A. triticina* [220]. Moreover, over 30 mycotoxins were isolated belonging to different classes based on chemical structure [221]. The genus *Alternaria* includes saprophytic, endophytic, and pathogenic species, and it is a cosmopolitan fungal genus found in natural and anthropogenic environments [220]. Among the *Alternaria* species, *A. alternata* is the most common in harvested fruit and vegetables, and it is the most important species producing mycotoxins [222]. AOH, AME, and ALT belong to dibenzo-α-pyrone derivatives, while ATXs belong to perylene quinone derivatives [223]. TeA belongs to tetramic acid derivatives with antibacterial and phytotoxic activities and acute toxicity for mice, chicken, and dogs, as well as hematological disorders in human [224].

According to the EFSA, among ATs, the most frequently studied are AOH, AME, and TeA [222]. Although most ATs exhibit only low acute toxicity, AOH and AME are the most toxic due to mutagenic, carcinogenic, cytotoxic, and genotoxic effects, with evidence from in vitro toxicological studies using bacterial and mammalian cells [225]. AOH is more genotoxic than AME in human carcinoma colon cells [224]. Currently, there are no regulatory limits or monitoring guidelines established for ATs in food worldwide. The risk assessment for *Alternaria* toxins entitled “Dietary exposure assessment to *Alternaria* toxins in the European population” for four of the known ATs, namely, AOH, AME, TeA, and TEN, with the highest exposure to AOH, AME, and TeA in “toddlers” and “other children”, was recently published by the EFSA. The TTC approach (toxicological concern threshold) was implemented by the EFSA as there is little or no toxicity data on ATs in order to assess the levels of concern for human health. For genotoxic ATs (AOH and AME), a TTC value of 2.5 ng/kg b.w./day was set, whereas, for non-genotoxic ATs (TeA and TEN), a TTC value of 1500 ng/kg b.w./day was set, and these exposure estimates are unlikely to be a concern for human health [44]. A major need for the assessment of exposure of humans and animals to potential health risks is the acquisition of additional toxicological data on the contamination of food and feed with ATs [222].

Substrate composition, temperature, pH, and a_w_ are the most important biotic and abiotic parameters affecting the biosynthesis of mycotoxins and, thus, the biosynthesis of ATs. In particular, a_w_ and pH affect most the biosynthesis of *A. alternata* [224]. Table 11 shows representative studies on the occurrence of ATs (μg/kg) in food samples worldwide during 2014–2019. 

The studies were conducted in dried fruits, in wheat and wheat-based products, in fresh and dried tomatoes, in juice samples, and in red wine. ATs that are the focus of most studies are AOH, AME, TEN, TeA, and ALT [223]. The occurrence of ATs is reported in various countries like Germany [223], Argentina [224], Canada [221,229], China [108], the Netherlands [230], and Italy [227]. ATs are detected in a large range of foodstuff commodities such as dried fruit [108], wheat, bran, flour [224,228,229,230], fresh and dried tomatoes [228], peppers [231], wine, vegetable juices, fruit juices [223], beer [232], cereal-based products such as rice and oat flake [233], sunflower seeds, and sunflower oil [222].

### 2.9. Patulin

Structurally, PAT is a heterocyclic lactone (4-hidroxi-4*H*-furo [3,2-c]piran-2(6*H*)-ona). It has a molecular weight of 154.12 g/mol and low volatility [233,234]. In total, 60 different fungal species such as *Penicillium expansum* (*P. leucopus*), *P. crustosum*, *P. patulum* (*P. urticae* and *P. griseofulvum*), and *A. clavatus* produce PAT, whereas *P. expansum* is the most common PAT producer [233]. The strain significantly determines the amount of patulin produced. Neurotoxicity, immunotoxicity, carcinogenesis, teratogenicity, and mutagenicity are acute and chronic effects exerted by patulin on cell cultures [235]. Patulin causes immunotoxic and neurotoxic effects in animals, and there is no clear evidence that it is carcinogenic to humans [224].

The EU, US Food and Drug Administration, and Chinese legislation all set the upper limit of 50 µg/L/kg patulin in apple and fruit juices [234]. For fruit juices, concentrated fruit juices such as reconstituted and fruit nectars, and spirit drinks, cider, and other fermented drinks derived from apples or containing apple juice, the EU established maximum levels of 50 µg/kg. For solid apple products, including apple compote and apple puree intended for direct consumption, the EU established maximum levels of 25 µg/kg. For apple juice and solid apple products, including apple compote and apple puree, for infants and young children and labeled and sold as such, the EU established maximum levels of 10 µg/kg [235]. The JECFA implemented a PMTDI of 0.4 mg/kg b.w./day for PAT [224] in 1995.

PAT is found in fruits and vegetables, especially apples and apple products in various parts of the world, and occasionally in other fruits such as pears, oranges, grapes, and their products [236,237,238,239,240]. If rotten fruits, especially apples, are not removed during fruit juice processing, patulin is transferred to juices [224]. PAT was initially studied as a potential antibiotic, but subsequent research showed human toxicity, including nausea, vomiting, ulceration, and hemorrhage [3]. The EU, US, and China present major problems of PAT contamination as they are the main producers of apples and apple products [234]. Table 12 presents representative studies on the occurrence of PAT (μg/kg or μg/L) in food samples worldwide during 2014–2019. The studies were conducted in dried fruits (figs, raisins), juices (apple and mixed fruit), and jam. 

## 3. Mycotoxin Control Strategies: Prevention and Decontamination/Detoxification in Foods

The reduction of mycotoxin contamination in agricultural commodities is a very important problem in many countries worldwide, which led to various preventive measures [11]. All pre-harvest strategies aim to avoid the development of toxigenic fungi and, hence, mycotoxins. However, once mycotoxins are produced, detoxification of foods should be based on post-harvest practices [248].

### 3.1. Pre-Harvest Strategies

Strategies for pre-harvest prevention include good agricultural practices (GAPs), good manufacturing practices (GMPs), appropriate environmental factors, and favorable storage practices [248]. GAPs include the implementation of a crop rotation program, use of registered insecticides, fungicides, and herbicides for control of insect damage, fungal infection, and weed eradication, proper treatment of the seed bed, soil analysis to determine the need to add fertilizers, and enhancement of genetic synthesis to suppress mycotoxin production [249,250]. Moreover, the use of biological control agents, such as antagonistic fungi, is an important pre-harvest strategy to prevent mycotoxin contamination in staple cereals, grapes, and apples [251,252]. At food processing plants, GMPs must be applied in conjunction with GAPs to act cooperatively with hazard analysis and critical control points (HACCP) [252]. Temperature and humidity exert the greatest influence on mycotoxigenic fungi for the production of mycotoxins, among the environmental factors. As it concerns favorable storage practices, temperature, moisture level, and humidity of warehouses are crucial factors for mold growth and mycotoxin production [248].

### 3.2. Post-Harvest Strategies

Decontamination/detoxification of mycotoxins from various agricultural products is a global problem, both scientific and practical. It was shown that mycotoxins can be eliminated by natural means such as thermal insulation, radiation treatment, and low-temperature plasma, chemical methods, such as oxidation, reduction, hydrolysis, alcoholysis, and absorption, and biological methods with the use of biological agents [253]. Chemical and physical detoxification methods have many limitations; they cause nutrient loss, are time-consuming and ineffective, and need expensive equipment. In comparison, biological methods proved to be more effective, more specialized, and more environmentally friendly [254].

#### 3.2.1. Physical Treatment

Various practices are used to remove mycotoxins naturally. Some of them are grading, sorting, and the removal of the obviously affected parts of a lot. Moreover, drying, washing, cleaning, segregation, milling, boiling, roasting, irradiation, extrusion, microwave heating, and peeling are used as physical treatments for mycotoxin decontamination. Implementing preventive post-harvest HACCP approaches can contribute to the problem of mycotoxin contamination [68,251].

##### Sorting

Undoubtedly, cleaning and sorting constitute the first step of natural disinfection. Techniques such as sorting might be regarded as superior methods since they pose no risk of producing degradable products [255]. Sorting and removal of decayed and poor-quality fruits can significantly reduce patulin levels in fruit products by up to 99% [248]. Total FBs decreased in percentage between 26% and 69% in maize after purification [255]. After sorting infected maize, a decrease of 27% to 93% FB was observed. Aflatoxin infection is usually heterogeneous; thus separation of damaged nuclei can effectively reduce infection. The use of ultraviolet radiation was also applied to reduce AFs in the sorting of cereals [256].

##### Processing

Processing techniques can reduce the concentration of mycotoxins, but they cannot completely destroy them [257]. The level of mycotoxin contamination can be reduced by softening, because the fungi accumulate on the surface of the granules. A study in Kenya showed a decrease in AFs in maize by peeling. The final flour was less contaminated, while mycotoxins DON and ZEN were detected on the surface of the granules at high levels. Temperature and time can affect the mycotoxin content of the final product. Although mycotoxins are thermally stable compounds, some conventional methods of preparing food (baking, frying) at temperatures above 100 °C may reduce certain mycotoxins. The processing temperature and moisture content of the granules affect the reduction of AFs by 50%–80% during the extrusion process [258]. Moreover, temperatures of 150–200 °C significantly reduced AFB1, causing 79% average reduction, being more effective at high humidity [259].

##### Storage

Storage conditions play an important role in controlling mycotoxins since they affect the overall growth of fungi. In particular, two main factors, temperature and high humidity, can promote both the fungal growth and the production of mycotoxins. Storage under controlled conditions, such as packaging practices, temperature control, ventilation, and appropriate air humidity, reduce the growth of fungi and the accumulation of mycotoxins [260]. Crop losses of 20% to 50% were recorded in developing countries due to inadequate storage practices [257].

##### Radiation

For many stored cereals, the use of natural detoxifying agents involves the use of radiation. Radiation is usually characterized as either ionizing radiation or non-ionizing radiation [258]. Radiation can reduce or eliminate pathogenic microorganisms, but it partially removes mycotoxins in foods. It can be applied on an industrial scale and is a technique that delivers energy and changes the molecular structure of food ingredients with a series of reactions [256].

Research showed that, in irradiated distilled water and fruit juices of orange, pineapple, and tomato infected with ZEA, ZEA toxicity was reduced and ZEA radiation was safe up to an irradiation of 10 kGy. A higher dose of radiation affected the quality of the fruit juices [261]. In a recent study by Luo et al. [68], after irradiation at 50 kGy with an electron beam in naturally infected corn to degrade ZEN and OTA, decreases of 71.1% and 67.9% were recorded. In addition, reduction of AFB1 greater than 95% (at 6 kGy) was achieved when gamma irradiation was used for rice processing [260]. Irradiation in apple juice for 5 min caused a significant decrease in PAT (83%) [233].

While radiation was proposed as a promising approach to mycotoxin detoxification, its effectiveness remains questionable because it can cause physical, chemical, and biological effects following potential molecular reactions [68].

##### Cold Plasma

Cold plasma (CP) has strong antimicrobial effects [256] and it is used in food processing to eliminate pathogens [258]. The fourth state of matter is the alternative name of plasma, mainly consisting of photons, ions, and free radicals such as reactive oxygen and nitrogen species with unique physical and chemical properties [262,263]. Cold atmospheric pressure plasma (CAPP) technology is a different technique, which is promising, low-cost, and environmentally friendly for the decontamination of mycotoxins [256,263]. Low-pressure cold plasma was used for detoxification of up to 50% of alfatoxins on the surface of nuts [264]. This technique requires cautious use as no research on the possible formation of toxic compounds was performed [265].

Significant reduction of AFB1 and FB1 mycotoxins of up to 66% was achieved in maize by the use of CAPP, after just 10 min of treatment [263]. In addition, the use of cold atmospheric plasma caused a 93% reduction in AFs, 90% reduction in TCs, 100% reduction in ZEA, and 93% reduction in FUs after 8 min of exposure [266]. In addition, plasma treatments of only 5 s caused 100% degradation of AFB1, DON, and NIV [6].

##### Mycotoxin Binders

Mycotoxin binders inhibit the absorption of mycotoxins as they bind to mycotoxins and do not allow their entry into the bloodstream from the gut. Various absorbent materials are activated carbon, aluminosilicates, complex non-digestible carbohydrates, and cholesterol [267]. The use of binding mycotoxins is an alternative physical technique [268] to the microbial degradation of AFs. Cleavage of the lactone ring is a potential target for microbial enzymes, and its cleavage reduces the toxicity of AFs [268]. According to research, to remove patulin from naturally infected cider, as well as to remove aflatoxin in naturally infected milk, activated carbon was used. Mycotoxin level was reduced, but more studies are needed to ensure food safety [256].

#### 3.2.2. Chemical Control

##### Bases (Ammonia, Hydrated Oxide)

Treatment of seeds with ammonia reduces a number of mycotoxins (AFs, FBs, OTs) to undetectable levels, while the growth of mycotoxigenic fungi is inhibited. Nevertheless, treatment with bases is forbidden in the EU for food intended for human consumption. The application of a mixture of glycerol and calcium hydroxide contributed significantly to mycotoxin detoxification [248]. Sodium hydroxide and potassium hydroxide are often used in the degradation of AFB1 in contaminated oil, although these chemicals can cause secondary contamination and have harmful effects on the nutritional value of the products [269].

##### Chitosan

Chitosan is a linear polysaccharide, second in abundance in nature after cellulose, inhibiting fungi, bacteria, and viruses. Biocompatibility and antimicrobial properties make chitosan very interesting for the preservation of foods [270,271]. The combined effects of chitosan and a_w_ for controlling the fungal growth and mycotoxin production of FBs and DON by the *Fusarium* species (*F. proliferatum*, *F. graminearum*, and *F. verticillioides*) on maize and wheat were reported, showing a decrease in DON and FB production in irradiated maize and wheat grains following the application of low-molecular-weight chitosan with deacetylation above 70%, and a dose of 0.5 mg/g [271]. In addition, the application of 1% chitosan enriched with 1% lemon essential oils in figs reduced the from marine brown algae *Ascophyllum nodosum* reduced the levels of DON in wheat grains [272].

##### Ozone Treatment

The use of ozone (O_3_) in the degradation of several mycotoxins was reported in many papers [273,274,275,276,277,278]. Ozonation is an easy technology, which does not leave harmful residues after application. Ozone is used to disinfect cereals, vegetables, and fruits, or to detoxify mycotoxins [278].

Ozone gas was reported by Agriopoulou et al. [279] to be particularly successful in the degradation of aflatoxins, mainly AFB1 and AFG1, since there is a C8–C9 double bond in their structures. Specifically, AFG1 proved to be the most sensitive. Ozone treatment under optimum conditions (55 g O_3_∙h^−1^ for 6 h) showed a significant decrease in DON (29%–32%) and its modified form DON-3-glucoside (DON-3-Glc) (44%). Moreover, significant microbial decline was observed in durum wheat, leaving chemical and rheological properties of semolina and pasta from ozonated wheat unaffected [273].

DON was transformed into 10 ozonized products (C_15_H_18_O_7_, C_15_H_18_O_9_, C_15_H_22_O_9_, C_15_H_20_O_10_, C_15_H_18_O_8_, C_15_H_20_O_9_, C_14_H_18_O_7_, C_14_H_16_O_6_, C_15_H_20_O_7_, and C_15_H_20_O_10_) after treatment with gaseous ozone [280]. DON degradation rate was positively correlated with ozone concentration and treatment time. Specifically, the rate of degradation of DON in solution reached 54.2%, for a treatment time of 30 s and an ozone concentration of 1 mg∙L^−1^. DON degradation was significantly influenced by the moisture content of the granules. The degradation rate of DON was 57.3% when ozone concentrations of 60 mg∙L^−1^ were applied for 12 h in wheat with a moisture content of 17.0% [278].

According to research by Li et al. [281], fresh noodles made from ozone-treated wheat flour retained more in relation to microbial growth.

#### 3.2.3. Biological Control

In the last 20 years, many researches from groups with different backgrounds and research experience made great achievements in the search for biological agents for mycotoxin detoxification [282]. The use of microorganisms such as bacteria, yeast, and fungi in the degradation of mycotoxins in food and feed is widely reported [21,254,283]. Detoxification/degradation of mycotoxins by biological means offers an alternative approach to the control of mycotoxins, since it can lead to the production of fewer or even no toxic intermediates and end products. The use of pure microbial strains greatly contributed to the disinfection of mycotoxins in vitro. Moreover, the effectiveness of fermentation in reducing and eliminating mycotoxins was also demonstrated [251].

##### Bacteria

Certain bacteria have the ability to bind mycotoxins in foods or liquids [21]. The only bacterium among the more than 1000 tested for possible degradation of AFs capable of irreversibly removing aflatoxin from solutions was *Flavobacterium aurantiacum* B-184. Detoxification of AFB1 through *Enterococcus faecium* is accomplished by binding to the cell-wall elements of the bacterium. Peptidoglycans and polysaccharides of bacterial cell walls were shown to be responsible for the binding of mycotoxins with the help of microorganisms [284].

Moreover, bacterial detoxification of mycotoxin DON evolved due to research efforts and advances. Aerobic oxidation and partitioning of this mycotoxin into C3 carbon carried by multiple species of *Devosia* provides solutions aimed at reducing DON contamination [282].

Lactic acid bacteria (*Lactobacillus* (L.) *casei* and *Lactobacillus reuteri*) proved effective in binding to AFs in aqueous solutions. In other in vitro tests, *Lactobacillus amylovorus* and *Lactobacillus rhamnosus* presented a binding efficiency of up to 60% AFB1, showing their potential to bind selected dietary contaminants [7]. Also, reductions 98% FB1 and 84% T-2 were demonstrated during the fermentation of whole-grain sorghum with *Lactobacillus fermentum* [285].

##### Yeast

The application of biological control agents (BCAs) is a promising strategy for the treatment of mycotoxin infection. The use of competing yeasts is of particular interest, since yeasts produce antimicrobial compounds with beneficial impacts on humans and animals; on the other hand they can develop rapidly on many substrates in bioreactors. In addition, unlike many filamentous fungi or bacterial antagonists, yeasts do not produce allergens or other secondary metabolites [286,287]. *Saccharomyces cerevisiae* is a probiotic yeast which can significantly degrade DON and reduce the rate of lactate dehydrogenase (LDH) release in DON-stimulated cells [288].

Moreover, low concentrations of mycotoxins AFB1 and OTA in chicken diets can be reduced with the addition *S. cerevisiae* yeast cell walls [289]. In addition, the effectiveness of reducing mycotoxin patulin by *S. cerevisiae* in fermented foods by increasing fermentation time and temperature was investigated. Yeast cells are capable of removing PAT via physical adsorption. In fact, the O-N/N-H protein and polysaccharide bonds of cell walls interact with PAT [290] *Kluyveromyces marxianus* were used to bind mycotoxins AFB1, OTA, or ZEA. The results showed that mycotoxins can bind to the cell membrane, especially to *C. utilis* [291]. In another study, the yeast *Yarrowia lipolytica* decreased the concentration of OTA to about half of the initial level introduced into the culture [241].

In addition, a yeast strain of *Rhodotorula mucilaginosa* (*R. mucilaginosa* JM19) was used to degrade PAT, and analysis was performed by HPLC-UV. The results showed that the degradation product of PAT was dexipitulic acid. The temperature, cell density, and initial concentration of PAT contributed greatly to the degradation of PAT through *R. mucilaginosa* JM19. After 21 h at 35 °C and when the density of yeast cells was above 1 × 10^8^ cells/L, a 90% decrease in PAT was observed. At an initial PAT concentration of 100 μg/mL, *R. mucilaginosa* JM19 was shown to be capable of causing more than 50% degradation, indicating its usefulness in the degradation of PAT in foods and raw materials [292].

##### Food Fermentation

The fermentation of foods improves their quality while granting them particularly desirable properties for consumers. Fermentation is a fairly inexpensive mycotoxin disinfection approach that can be used both to improve the ingredients in foods and to reduce and even eliminate mycotoxins. Fermentation can be an alternative and desirable technique to reduce mycotoxins compared to costly and impractical techniques. The nature of metabolites and the toxicity of products formed after fermentation should be carefully documented in order to produce safe foods [251].

##### Fungi

The use of non-toxic strains of *A. flavus* and *A. parasiticus* on maize, cotton, pistachio, and peanuts yielded remarkable success in reducing aflatoxin contamination. Regarding the fungi and their detoxification, it was reported that fungi capable of producing aflatoxins could also break them down. This is because these fungi are often able to degrade and possibly convert and use degradation products as a source of energy under starvation conditions [7]. Fungi such as *Aspergillus*, *Rhizopus, Trichoderma, Clonostachys,* and *Penicillium* spp. show efficient abilities in the detoxification of mycotoxins [250]. In both west and east Africa, the biocontrol of AFs in maize with non-toxigenic microbial strains is based on competition. Specifically, large amounts of non-toxic inoculants of *A. flavus* and *A. parasiticus* enter the soil around the crops and compete with toxigenic strains [252].

#### 3.2.4. Enzymatic Detoxification

The enzymatic detoxification of mycotoxins combines the characteristics of chemical and biological processing. It has high performance and specialization, with application under mild conditions, and it does not cause toxicity to organisms. In addition, enzymes as catalysts are involved in non-stoichiometric ratios of mycotoxins [253]. Some *Aspergillus* species can produce an enzyme that is naturally capable of detoxifying fumonisins, including those produced by *Fusarium* [293]. The activity of enzymes such as β-1,3-glucanase and chitinase against pathogens may vary depending on the characteristics of the microorganism. The delay and decrease in growth of fruit spoilage fungi are affected by the application of β-1,3-glucanases and chitinases [294]. Inhibition of *Penicillium simplicissimum*, *A. Niger* complex, *Penicillium nalgiovense*, and *A. flavus* growth on salami surface samples was induced by spraying β-glucanase at 50% and chitinase at 50% and 40% concentrations. Therefore, β-glucanase and chitinase may be a safe alternative for the fermented sausage industry to control fungal spoilage [294]. Moreover, microbial manganese peroxide, oxidase enzymes, catalase, and laccase enzymes were used for the enzymatic detoxification of AFB1 [252,258]. However, enzymes have an unexplored profile when detoxifying food contaminants due to their favorable toxicology and specialization. In the EU, no enzyme is approved for the removal of mycotoxin contamination from foodstuffs [258].

#### 3.2.5. Novel Detoxification Strategies

##### Nanoparticles

Many papers proposed the removal of mycotoxins using the promising adsorbents of nanoparticles. Magnetic carbon nanocomposites were used for AFB1 detoxification, chitosan-coated Fe_3_O_4_ nanoparticles were reported for PAT decontamination, and silver nanoparticles were reported for degradation of *Fusarium* spp. and their main associated mycotoxins [248,295]. According to a recent study, a new photocatalyst nanoparticle UCNP@TiO_2_ (upconversion nanoparticle) was synthesized and used to degrade DON. The results showed a decrease of DON in cereal products below the permissible limits (1 ppm) after 90 min and total degradation after 120 min of illumination. The UCNP@TiO_2_ composite material was efficient and green, and the degradation products were only slightly toxic or even non-toxic. Therefore, this degradation technology can be used for mycotoxin detoxification [296]. González-Jartín et al. [297] reported elimination of up to 87% of mycotoxins from nanocomposites composed of mixtures of activated carbon, bentonite, and aluminum oxide.

##### Plant Extracts

Different essential oils (EOs) and their main bioactive compounds were used for the antifungal and antimycotoxigenic properties [298,299,300], and they were demonstrated to inhibit the production of some mycotoxins [273]. The use of botanicals is usually preferable in the removal of toxigenic fungi and mycotoxins compared to chemical treatments because they are considered safe to humans and environmentally friendly. Several researchers reported that the oil of clove and its major ingredient, eugenol, as well as the turmeric essential oil, inhibit *Aspergillus* growth and AFB1 production. The growth of *A. flavus* and *P. citrinum* and their toxins were inhibited by the application of whole clove in culture media and rice grains [248,301].

A recent scientific study demonstrated the effect of the Spanish paprika smoker “Pimentón de la Vera” on the development of *A. parasiticus* and *P. nordicum* and the production of AFB1, AFG1, and OTA. The addition 2%–3% Spanish paprika smoker in meat products such as fillets or sausage preparations helped minimize the development and production of mycotoxins AF and OTA [302]. Moreover, capsaicin, a natural compound, inhibited OTA production in grapes by *Aspergillus* section *Nigri* strains from 28.9% to 78.1%, and by *A. carbonarius* of 61.5% [303].

## 4. Conclusions

Mycotoxins are among the most prominent and dangerous toxins associated with food safety. They attract worldwide attention because they are important contaminants with a multitude of effects on both human and animal health, causing significant economic problems throughout the agri-food chain. High-quality and safe foods can emerge if HACCP is implemented across the food chain from farm to fork and at all stages of food handling, providing global food security. Prevention is an important strategy in the fight against mycotoxins and should be applied in the pre-harvest stages, in raw materials and processed foods. Detoxification methods can also be applied without affecting the organoleptic characteristics of the foods. Finally, continuous monitoring of mycotoxins in food products can prevent significant problems in the marketing, distribution, and consumption of foods.

## Figures and Tables

**Table 1:** Most important mycotoxins and the United States (US) and European Union (EU) limits on food and animal feed levels. FDA—Food and Drug Administration; IARC—International Agency of Research on Cancer.

Mycotoxin	IARC Number *	Acronym	Fungal Species	Food Commodity	US FDA (µg/kg)	EU (µg/kg)	References
Aflatoxins B1, B2, G1, G2	1*	AFB1 AFB2 AFG1 AFG2	*Aspergillus flavus, Aspergillus parasiticus*	Maize, wheat, rice, peanut, sorghum, pistachio, almond, ground nuts, tree nuts, figs, cottonseed, spices	20 for total	2–12 for B1 4–15 for total	[37]
Aflatoxin M1	2B*	AFM1	Metabolite of aflatoxin B1	Milk, milk products, meat	0.5	0.05 in milk 0.025 in infant formulae and infant milk	[37]
Ochratoxin A	2B*	OTA	*Aspergillus ochraceus, Aspergillus carbonarius Penicillium verrucosum, Penicillium nordicum*	Cereals, dried vine fruit, wine, grapes, coffee, cocoa, cheese	Not set	2–10	[37]
Fumonisins B1, B2, B3	2B*	FB1 FB2 FB3	*Fusarium verticillioides, Fusarium proliferatum*	Maize, maize products, sorghum, asparagus	2000–4000	200–4000	[38]
Zearalenone	3*	ZEN	*Fusarium graminearum (F. roseum),* *Fusarium culmorum Fusarium equiseti,* *Fusarium cerealis, Fusarium verticillioides,* *Fusarium incarnatum*	Cereals, cereal products, maize, wheat, barley	Not set	20–100	[38]
Trichothecenes (type B: deoxynivalenol)	3*	DON	*Fusarium graminearum, Fusarium culmorum,* *Fusarium cerealis*	Cereals, cereal products	1000	200–50	[38,39]
Patulin	3*	PAT	*Penicillium expansum* *Bysochlamis nívea, Aspergillus clavatus,* *Penicillium patulum Penicillium crustosum*	Apples, apple juice, and concentrate, pears, peaches, grapes, apricots, olives low acid fruit juices	50	10–50	[37,40]
Trichothecenes (type A: HT-2)	3*	HT2	*Fusarium langsethiae, Fusarium sporotrichioides*	Maize, wheat, barley, oat, rye	15	25–1000	[41]
Trichothecenes (type A: T-2 toxin)	3*	T-2	*Fusarium langsethiae,* *Fusarium sporotrichioides*	Maize, wheat, barley, oat, rye	15	25–1000	[41]
Enniatins		ENNs	*Fusarium tricinctum, Fusarium avenaceum*	Corn	Not set	Not set	[21]
Ergot alkaloids		EAs	*Claviceps purpurea, Claviceps fusiformis,* *Claviceps africana, Neotyphodium spp*	Rye, rye-containing commodities, wheat, triticale, barley, millet and oat	Not set	Not set	[19,42]
Alternariol		AOH	*Alternaria alternata*	Grain and grain-based products, vegetables and vegetable products, fruits and fruit products, wine and beer, oilseeds and vegetable oils	Not set	Not set	[43]

IARC number definitions: 1, the mycotoxin is carcinogenic to humans; 2B, the mycotoxin is possibly carcinogenic to humans; 3, the mycotoxin is not classifiable as to its carcinogenicity to humans.

**Table 2:** Mycotoxin notification in the EU during the decade 2009–2018 (Rapid Alert System for Food and Feed (RASFF), Annual Report, 2009–2018) [23,24,25,26,27,28,29,30,31,32].

Mycotoxins	2009	2010	2011	2012	2013	2014	2015	2016	2017	2018
Aflatoxins	638	649	585	484	341	314	438	348	529	536
Deoxynivalenol (DON)	3	2	11	4	8	6	11			
Fumonisins	1	3	4	4	7	3	5			
Ochratoxin A	27	34	35	32	54	38	42	141		33
Patulin							2			
Zearalenone				4		3	2			
Total	669	688	635	525	410	364	500	489	529	569

**Table 3:** Notifications by hazard category in the EU in 2018 (RASFF, Annual Report, 2018) [32]. GMO—genetically modified organism.

Hazard Category	Alert	Border Rejection	Information for Attention	Information for Follow-Up
Allergens	158	11	35	3
Biological contaminants (other)	22	4	20	
Food additives and flavorings	19	64	24	35
Foreign bodies	106	10	19	33
GMO food or feed		9	1	3
Novel food	8	6	15	22
Metals	56	13	55	9
Industrial contaminants				1
Mycotoxins	88	508	55	4
Parasitic infestation		1	17	23
Pathogenic microorganisms	349	302	191	137
Pesticides residues	48	154	60	14
Residues of veterinary medicinal products	10	15	15	8

**Table 4:** Representative studies on the occurrence of aflatoxin (AF) distribution (μg/kg) in food samples around the world during 2014–2019.

Country	Food Matrix	AFs	*N* Samples	Incidence %	Mean μg/kg	Range μg/kg	Detection Technique	Reference
China	Raw milk	AFM1	530	52.8	0.07	0.01–0.2	LC-MS/MS	[82]
Nigeria	Breast milk	AFM1	40	77	0.066	0.001–0.601	HPLC	[83]
Burkina Faso	Infant formula	AFB1	199	84	3.8	0–87.4	HPLC	[84]
China	Wheat	AFB1	348	0.28	7.3	<0.10–7.3	LC-MS/MS	[85]
		AFB2	348	0.28	1.2	<0.10–1.2		
		AFG1	348	0	−	−		
		AFG2	348	0	−	−		
China	Rice	AFB1	370	63.50	0.6	0.03–20.0	HPLC	[86]
		AFB2	370	17.57	0.2	<0.1–3.2		
China	Maize	AFB1	44	2.3	148.4	−	HPLC	[87]
Turkey	Walnut	AFs	48	44	1.33	0.58–15.2	HPLC	[88]
Turkey	Seedless black raisins	AFs	25	64	0.4	0.02–2.07	HPLC	[89]
	Dried figs		45	51	1.78	0.16–5.20		
	Ground red peppers		25	72	2.30	0.04–3.47		
	Walnuts without shell		25	64	1.68	0.66–2.62		
Nigeria	Ginger	AFs	120	81	3.13	0.11–9.52	HPLC	[90]
Egypt	Dried date palm	AFB1	28	4	−	14.4	LC-MS/MS	[77]
		AFB2				2.44		
Tanzania	Sun-dried sweet potato chips	AFs	80	36	40.31	10.49–75.12	HPLC	[91]
Ghana	Cereal-based products	AFs	50	72	−	0.18–25.93	HPLC	[56]
Czech Republic	Barley and malt samples	AFs	52	Nd ^a^	−	−	LC-MS/MS	[92]

^a^ Nd—not detected.

**Table 5:** Representative studies on the occurrence of ochratoxin A (OTA) distribution (μg/kg or μg/L) in food samples around the world during 2014–2019.

Country	Food Matrix	*N* Samples	Incidence %	Mean μg/kg	Range μg/kg	Detection Technique	Reference
China	Dried vine fruits	56	58.9	0.99	<0.07–12.83	HPLC	[107]
China	Dried fruits	220	5	1.9	0.2–8.8	LC-MS/MS	[108]
China	Rice	370	4.87	0.9	0.30–3.2	HPLC	[86]
Burkina Faso	Infant formula	199	7.5	0.1	0–3.2	HPLC	[84]
Syria	Fruit-based products	12	33	0.093	0.019–0.156	HPLC	[109]
	Cereal-based baby food	30	43	0.094	0.02–0.329		
Brazil	Breast milk	86	0	−	−	LC-MS/MS	[110]
Turkey	Eggplant	50	100	17.67	8.88–21.35	HPLC	[111]
	Green bell pepper			20.53	15.38–24.70		
Egypt	Dried date palm	28	11	58.7	1.48–6070	LC-MS/MS	[77]
Lebanon	Spices	94	30	7.1	2–33.9	LC-MS/MS	[74]
Czech Republic	Barley and malt samples	52	Nd ^a^	−	−	LC-MS/MS	[92]
Syria	Durum wheat	40	30	0.6	0.4–0.7	LC-MS/MS	[112]
China	Peanut	28	7.1	NA ^b^	0.25–0.65	HPLC	[113]
Qatar	Baby food, cereal based	51	31	NA ^b^	0.05–≥0.50	LC-MS/MS	[114]
Kingdom of Saudi Arabia	Cardamom	80	48	NA ^b^	30–78	HPLC	[115]
Portugal	Roasted coffee	6	33	1.84 μg/Kg	LOD–10.31	HPLC	[116]
	Ground roasted coffee	5	20	1.45 μg/Kg	LOD–7.26		
Denmark, Kenya, Tanzania, Uganda	Roasted coffee	57	46	NA ^b^	2.3	UHPLC-MS/MS	[117]

^a^ Nd—not detected. ^b^ NA—not available in the publication.

**Table 6:** Representative studies on the occurrence of fumonisin (FU) distribution (μg/kg) in food samples around the world during 2014–2019.

Country	Food Matrix	Fumonisins	*N* Samples	Incidence %	Mean μg/kg	Range μg/kg	Detection Technique	Reference
China	Maize	FB1	44	100	116.5	16.5–315.9	HPLC	[89]
Burkina Faso	Infant formula	FB1 + FB2	199	1.5	30.3	0–672.9	HPLC	[86]
United States	Infant cereal-based products	FB1	64	2	NA	<LOD–6.2	LC-MS/MS	[136]
		FB2		8		<LOD–15.8		
Egypt	Dates palm	FB2	28	7	10.6	4.99–16.2	LC-MS/MS	[77]
Serbia	Crop maize	FUs	90	100	1730	520.0–5800.0	ELISA	[137]
China	Corn flakes	FB1	14	100	104.1	1.0–171.0	UPLC-MS-MS	[138]
		FB2	14	93	14.2	<0.27–25.6		
		FB3	14	93	17.3	<0.27–31.5		
Lebanon	Spices	FB1	94	64	6432.3	18.2–113474.5	LC-MS/MS	[74]
		FB2		35	230.2	15.1–1757.4		
Argentina	Wheat-based products	FB1 + FB2	91	74	1.54	0.05–18.9	HPLC-MS/MS	[132]
China	Wheat flour	FB1	369	6.2	NA	0.3–34.6	UPLC-MS/MS	[138]
Tanzania	Sun-dried sweet potato chips	FB1	80	97.5	44.69	29.34–628.78	HPLC	[91]
Brazil	Wheat cultivars	FB1	11	54	2814.33	958–4906	HPLC	[139]
Argentina	Wheat	FB1	135	97	30.07	0.16–680.4	HPLC-MS/MS	[133]
	Durum wheat		40	77.5	84.75	0.15–1304.4		
	Wheat	FB2	135	51	2.88	0.25–23.7		
	Durum wheat		40	42.5	10.47	0.43–47		
Italy	Durum wheat	FB1	4	100	33	15–51	HPLC-MS/MS	[140]
Syria	Durum wheat	FB1	40	10	5	5–6	HPLC-MS/MS	[112]

^a^ NA—not available in the publication.

**Table 7:** Representative studies on the occurrence of trichothecene (TC) distribution (μg/kg) in food samples around the world during 2014–2019.

Country	Food Matrix	TCs	*N* Samples	Incidence %	Mean μg/kg	Range μg/kg	Detection Technique	Reference
China	Wheat	DON	38	100	106.5	0.5–604.0	LC-MS/MS	[157]
China	Maize	DON	44	65.9	831.0	5.8–9843.3	HPLC	[87]
Romania	Wheat	DON	31	26	748	110–1787	LC-MS/MS	[158]
	Flour		35	3	190	190		
Pakistan	Rice	DON	180	8	6.99	<LOD	LC-MS/MS	[159]
Brazil	Wheat	DON	48	100	2398	1329–3937	HPLC	[160]
Italy	Wheat	DON	293	97	NA ^a^	56–27088	GC-MS	[161]
Brazil	Wheat	DON	53	47	641	243–2281	HPLC	[162]
China	Wheat	DON	348	91	240	240–1129	LC-MS/MS	[85]
Iran	Wheat	DON	96	83	630	23–1270		[163]
Finland	Wheat	DON	30	NA ^a^	866	NA–5510	LC-MS/MS	[164]
Brazil	Wheat	DON	58	91	360	NA–1310	HPLC	[165]
Brazil	Wheat	DON	745	86	1690	NA–8501	HPLC	[166]
					407	NA–2419		
Brazil	Wheat	DON	150	99	706	183–2150	HPLC	[167]
Hungary	Wheat	DON	29	72	NA ^a^	NA–1880	ELISA	[168]
Argentina	Wheat	DON	84	100	1750	NA–9480	LC-MS/MS	[169]
Switzerland	Wheat	DON	686	80	607	NA–10600	LC-MS/MS	[170]
Brazil	Wheat	DON	172	77	234	73–2794	HPLC	[171]
Poland	Wheat	DON	92	83	140	10–1265	HPLC	[172]
China	Wheat	DON	181	82	500	33–3030	HPLC	[173]
Hungary	Maize	DON	29	86	1872	225–2963	ELISA	[168]
		T-2	29	55	69	NA–146		
Serbia	Corn flour	DON	56	42.9	101	NA–931	HPLC	[174]
	Cornflakes		15	40	255	NA–878		
Brazil	Barley	DON	76	94	310–15,500	1700–7500	LC-MS/MS	[175]
Brazil	Bakery Products	DON	36	100	591	60–1720	HPLC	[165]
India	Infant Foods	DON	29	66	NA ^a^	5–228	ELISA	[176]
Germany	Noodles and Pasta	DON	40	97	387	60–1609	HPLC	[165]
Finland	Oat	DON	31	100	NA ^a^	23,800	LC-MS/MS	[164]
UK	Oat	DON	303	32	28	NA–1866	LC-MS/MS	[177]
Finland	Oat	DON	1672	79	NA ^a^	NA–21608	GC-MS	[178]
Poland	Oat	DON	147	31	NA ^a^	NA–2975	HPLC-HRMS	[179]
Tunisia	Cereal-based products	HT-2	32	3	1	<LOD–209	LC-MS/MS	[153]
Lebanon	Wheat grains, wheat flour, and bread	HT-2	137	0	−	−	LC-MS/MS	[180]
		T-2						
Spain	Cereal-based food, beer	HT-2	479	NA ^a^	0.047–0.214	NA ^a^	GC-ECD	[181]

		T-2	479	0.006	0.215–0.072	NA ^a^		
Spain	Wheat semolina	HT-2	15	33.3	8.9	6.7–15.2	GC–QqQ-MS/MS	[182]

^a^ NA—not available in the publication.

**Table 8:** Representative studies on the occurrence of ZEN distribution (μg/kg) in food samples around the world during 2014–2019.

Country	Food Matrix	N Samples	Incidence%	^a^ Mean μg/Kg	^a^ Range μg/Kg	Detection Technique	Reference
China	Wheat	348	13.2	8.4	<0.25-98.8	LC-MS/MS	[85]
China	Maize	50	94	109.1	0.2-3613.0	LC-MS/MS	[192]
China	Maize	44	13.6	50.8	40.7-1056.8	HPLC	[87]
Hungary	Maize	29	41	267	NA^a^-565	ELISA	[168]
South Korea	Infant formula	36	25	NA^a^	3.3-17.6	UHPLC	[193]
Serbia	Crop maize	90	0	-	-	ELISA	[137]
Pakistan	Rice	180	15	8.48	NA^a^	LC–MS/MS	[159]
Lebanon	Spices	94	30	30.6	0.4-305.4	LC-MS/MS	[74]
Brazil	Wheat flour	39	2.6	-	26.7	UPLC–MS/MS	[194]
China	Peanut	28	36	NA^a^	0.09–26.8	HPLC	[113]
China	Grapes	36	6	NA^a^	0.29-0.36	UHPLC–MS/MS	[75]
	Wine	42	7	NA^a^	<LOQ to 1.85		
Syria	Wheat	40	25	13	4-34	LC–MS/MS	[112]
Italy	Wheat	47	34	44	7-230	HPLC–MS/MS	[112]
Qatar	Baby food, milk based	51	37	NA	1 - ≥5	HPLC	[114]

^a^ NA: Not available in the Publication.

**Table 9:** Representative studies on the occurrence of emerging *Fusarium* mycotoxins distribution (μg/Kg) in food samples around the world during 2014–2019.

Country	Food Matrix	Emerging *Fusarium* mycotoxins	N Samples	^a^ Incidence %	Mean μg/Kg	^b^ Range μg/Kg	Detection Technique	Reference
Serbia	Maize	FP	21	76	-	85.4–1121	LC-MS/MS	[196]
Japan	Corn	BEA	44	34	3.8	NA^b^-26.1	LC-MS/MS	[202]
	Wheat flour		66	Nd^a^	-	-		
	Wheat flour	ENB	66	81.8	33.4	NA^b^-237		
Italy	Wheat	BEA	43	14	0.0004	0.0018-0.0051	HPLC–MS/MS	[112]
Syria	Wheat	BEA	40	13	0.0002	0.0015-0.0017	HPLC–MS/MS	[112]
Italy	Wheat	BEA	54	67	-	0.04-5.28	LC-MS/MS	[211]
Italy	Durum wheat	BEA	108	94	-	0.03-56.40	LC-MS/MS	[211]
Morocco	Wheat	BEA	80	10	0.9	NA^b^-16	LC-MS/MS	[205]
		ENA		14	6.8	NA^b^-75		
		ENA1		18	13	NA^b^-134		
		ENB		61	57	NA^b^-2570		
		ENB1		53	27	NA^b^-345		
Serbia	Maize	ENNs	29	10	-	0.12–0.47	LC-MS/MS	[212]
Italy	Wheat	ENA	43	14	0.0012	0.0031-0.0181	HPLC–MS/MS	[112]
Syria	Wheat	ENA	40	10	0.0002	0.0015-0.0022	HPLC–MS/MS	[112]

^a^ Nd: Not detected. ^b^NA - Not available in the Publication.

**Table 10:** Representative studies on the occurrence of Ergot alkaloids distribution (μg/Kg) in food samples around the world during 2014–2019.

Country	Food Matrix	N Samples	^a,b^ Incidence%	^b^ Mean μg/Kg	Range μg/Kg	Detection Technique	Reference
Albania	Wheat	35	48.6	337.2	17.3–975.4	LC-MS/ MS	[215]
Canada	Barley	67	73	1150.50	2.21-29424.6	LC-MS/ MS	[214]
Italy	Rye-based products	16	85	NA^b^	2.6–188.6	LC-MS/MS	[42]
	Wheat-based products	55			2.5– 1142.6		
China	Cereal samples	123	Nd ^a^	-	-	LC-MS/MS	[219]
Canada	Cereals	228	NA ^b^	NA^b^	65-1140	LC-MS/MS	[216]

^a^ Nd: Not detected. ^b^ NA: Not available in the Publication.

**Table 11:** Representative studies on the occurrence of Alternaria toxins distribution (μg/kg or μg/L) in food samples around the world during 2014–2019.

Country	Food Matrix	Alternaria Toxins	N Samples	^a^Incidence %	^b^Mean μg/Kg	Range μg/Kg	Detection Technique	Reference
China	Dried fruits	AOH	220	2.3	12.0	3.5–27.4	LC-MS/ MS	[108]
		TeA		42.7	465.5	6.9–5665.3		
		AME		8.2	3.0	0.2–15.0		
		TEN		20.5	120.5	1.4–1032.6		
China	Wheat	TeA	370	100	289.0	6.0–3330.7	LC-MS/ MS	[226]
		AOH	370	47	12.9	1.3–74.4		
		AME	370	15	9.1	0.3–54.8		
Argentina	Flour	TeA	23	61	7360	LOQ-17,719	HPLC-DAD	[224]
	Wheat		21	57.1	19,190	LOQ-92,000		
	Bran		21	66.7	16,760	LOQ-82,600		
Italy	Dried tomatoes	TeA	10	100	16,291	425–81,592	LC-MS/MS	[227]
	Fresh tomatoes		8	100	1,010	11–4560		
Germany	Juice samples	TeA	88	62	NA^b^	1.4–19.2	LC-MS/MS	[223]
	Red wine	AME	11	Nd^a^	-	-		
China	Wheat flour	TeA	181	99.4	88.4	1.76–520	UPLC-MS/MS	[228]
	Dried noodle		52	96.2	47.6	4.86–158		
	Bread		50	98	11.7	1.95–38.2		
	Steamed bread		40	100	21.2	6.56–46.3		

^a^ Nd: Not detected. ^b^ NA: Not available in the Publication.

**Table 12:** Representative studies on the occurrence of PAT distribution (μg/kg or μg/L) in food samples around the world during 2014–2019.

Country	Food Matrix	N Samples	^a^Incidence %	^a^Mean μg/kg	Range μg/kg	Detection Technique	Reference
China	Dried fig	20	65.0	-	15.0-276.9	HPLC	[240]
	Raisins and other dried fruits	36	8.3	-	12.5-68.0		
	Juice	20	10.0	-	8.0-16.8		
	Jam	20	30.7	-	8.6-11.0		
China	Dried fruits source	220	0.5	30.6	30.6	LC-MS/ MS	[108]
China	Apple juice	23	39.1	1.0	0.50-4.82	LC-MS/ MS	[241]
	Mix fruit juice	20	20	0.74	0.50-2.46		
Qatar	Fruit-based products	13	100	20.8	1.02-61.3	LC-MS/MS	[242]
	Apple juice	20	100	NA^a^	5.27-82.21		
Tunisia	Fruit-based products	214	50	89	2-889	HPLC	[243]
Pakistan	Apple	36	75	NA^a^	<LOD–630.8	HPLC	[239]
China	Apple juice (including organic and conventional apple juice and juice concentrate)	4	NA^a^	NA^a^	<LOD–16.8	HPLC	[240]
Serbia	Apple juice (including organic and conventional apple juice and juice concentrate)	73	74	NA^a^	<LOD–65.4	HPLC	[244]
Argentina	Apple juice (including organic and conventional apple juice and juice concentrate)	4634	40	26	<LOD–19,622	HPLC	[245]
Malaysia	Apple juice (including organic and conventional apple juice and juice concentrate)	13	8	NA^a^	<LOD–26.9	HPLC	[246]
Italy	Products for babies (including apple juice, apple sauce, and compotes)	26	0	-	-	HPLC	[247]

^a^ NA: Not available in the Publication.
